# Variable effects on growth and defense traits for plant ecotypic differentiation and phenotypic plasticity along elevation gradients

**DOI:** 10.1002/ece3.4999

**Published:** 2019-02-27

**Authors:** Moe Bakhtiari, Ludovico Formenti, Veronica Caggìa, Gaëtan Glauser, Sergio Rasmann

**Affiliations:** ^1^ Institute of Biology University of Neuchâtel Neuchâtel Switzerland; ^2^ Institute of Plant Science University of Bern Bern Switzerland; ^3^ Neuchâtel Platform of Analytical Chemistry University of Neuchâtel Neuchâtel Switzerland

**Keywords:** common garden, ecotypic differentiation, elevation gradients, phenotypic plasticity, plant defense, secondary metabolites

## Abstract

Along ecological gradients, phenotypic differentiation can arise through natural selection on trait diversity and magnitude, and environment‐driven plastic changes. The magnitude of ecotypic differentiation versus phenotypic plasticity can vary depending on the traits under study. Using reciprocal transplant‐common gardens along steep elevation gradients, we evaluated patterns of ecotypic differentiation and phenotypic plasticity of several growth and defense‐related traits for two coexisting but unrelated plant species, *Cardamine pratensis* and *Plantago major*. For both species, we observed ecotypic differentiation accompanied by plasticity in growth‐related traits. Plants grew faster and produced more biomass when placed at low elevation. In contrast, we observed fixed ecotypic differentiation for defense and resistance traits. Generally, low‐elevation ecotypes produced higher chemical defenses regardless of the growing elevation. Yet, some plasticity was observed for specific compounds, such as indole glucosinolates. The results of this study may suggest that ecotypic differentiation in defense traits is maintained by costs of chemical defense production, while plasticity in growth traits is regulated by temperature‐driven growth response maximization.

## 1 INTRODUCTION

Species with wide distributions tend to exhibit large intraspecific variation in most functional and phenotypic traits. This geographical variation in biotic and abiotic factors across species distributions can lead to the evolution of morphologically and functionally different ecotypes (Hufford & Mazer, 2003; Kawecki & Ebert, 2004; Savolainen, Pyhäjärvi, & Knürr, 2007). Ecotypes are genetically distinct populations of a given species, displaying phenotypic traits that maximize fitness within a particular local abiotic and biotic conditions (Kawecki & Ebert, 2004). Along environmental gradients, trait‐mediated local adaptations of plant ecotypes are the result of selection for fitness maxima under local conditions (Gratani, Meneghini, Pesoli, & Crescente, 2003; Van Tienderen, 1989; Wadgymar, Daws, & Anderson, 2017). Such phenotypic differentiation can be produced by natural selection on specific loci responsible for the diversity and magnitude of traits (i.e. genotypic differentiation), or through phenotypic plasticity.

Phenotypic plasticity refers to the ability of a single genotype to produce different phenotypes under varying environmental conditions. Plasticity itself can also be selected for and evolve independently for different developmental, physiological, and reproductive traits, or in different habitats, to optimize organisms’ performance (Bradshaw, 1965; Gotthard, Nylin, & xf, and ren., 1995; Lortie & Aarssen, 1996; Murren et al., 2015; Scheiner, 1993; Sultan, 1987, 2003). Species with greater adaptive plasticity may be better equipped to survive in novel environments; facilitating their rapid geographical expansion into a broad range of environmental conditions (Baker, 1974; Oliva, Martínez, Collantes, & Dubcovsky, 1993; Spencer, Teeri, & Wetzel, 1994), ultimately promoting local adaptation (Baldwin, 1896; Ghalambor, Mckay, Carroll, & Reznick, 2007; Price, Qvarnström, & Irwin, 2003).

Being sessile organisms, plants should face stronger pressures leading to local adaptation. For instance, when moving from low to high latitudinal or elevational ranges, plant species or ecotypes tend to adapt by producing smaller seeds, to have earlier phenology, growing slower, and displaying greater investment in clonal reproduction (e.g. Chapin & Chapin, 1981; Körner, 2003; Moles et al., 2007; Montague, Barrett, & Eckert, 2008; Pilon, Santamarìa, Hootsmans, & Vierssen, 2003). Additionally, at the community level, interspecific interactions between species along biogeographical gradients are also expected to form clines. Since the initial Dobzhansky's postulation of a potential correlation between the strength of biotic interactions and the values of traits mediating interactions (Dobzhansky, 1950), there has been a great deal of interest in plant‐herbivore interaction along latitudinal gradients (Bolser & Hay, 1996; Coley & Aide, 1991; Schemske, Mittelbach, Cornell, Sobel, & Roy, 2009). A key prediction from these studies was that increased herbivory pressure at lower (tropical) latitudes compared to higher (temperate) latitudes should favor the evolution of more potent defenses in tropical plants (Coley & Barone, 1996; Moles et al., 2011; Pennings, Siska, & Bertness, 2001; Rasmann & Agrawal, 2011; Siska, Pennings, Buck, & Hanisak, 2002; Woods, Hastings, Turley, Heard, & Agrawal, 2012).

More recently, the same concepts have been applied to elevational gradients (Rasmann, Alvarez, & Pellissier, 2014). A decrease in species’ diversity at high versus low‐elevations can also be associated with a reduction in species interactions, which would lead to a relaxation of plant defenses at high elevation (Rasmann, Pellissier, Defossez, Jactel, & Kunstler, 2014). This has been observed at the community level (Callis‐Duehl, Vittoz, Defossez, & Rasmann, 2017; Descombes et al., 2016; Kergunteuil, Descombes, Glauser, Pellissier, & Rasmann, 2018), interspecific level (Defossez, Pellissier, & Rasmann, 2018; Pellissier et al., 2012) and intraspecific level (Pellissier, Roger, Bilat, & Rasmann, 2014; Scheidel & Bruelheide, 2004; Zehnder et al., 2009). The study of plant adaptation and species interactions along elevational clines comes with several advantages compared to studies along latitudinal gradients (Körner, 2007). In particular, plant adaptation to habitat‐specific abiotic and biotic factors can be studied along elevational transects with homogenous macroclimatic conditions, minimizing the effect of biogeographical history and barriers to gene flow (Rasmann, Pellissier et al., 2014; Sundqvist, Sanders, & Wardle, 2013).

Plant growth and defense related traits have been shown to vary in response to different abiotic and biotic conditions. Therefore, it is expected that biogeographical gradients should select for clinal adaptation in such traits (Woods et al., 2012). Furthermore, growth and defense traits can be subjected to resource allocation trade‐offs, and the correlated expression of these traits should serve to maximize plant fitness within a given herbivory and climatic environment (Agrawal, Conner, & Rasmann, 2010). For instance, high and low‐elevation *Plantago lanceolata *ecotypes growing at two temperature regimes (12 and 20°C to simulate cold and warm environment of different elevation gradients) showed strong plasticity in growth (i.e. both genotypes grew similarly within each environment), while their resistance to generalist herbivores reflected genetically‐fixed patterns; high‐elevation ecotypes were always less resistant, independently of the temperature regimes (Pellissier et al., 2014). Such differences would suggest that ecotypes growing at high elevation were selected to produce lower amounts of constitutive defenses because of lower amount of herbivory, while retaining a high degree of plasticity of growth‐related responses to temperature. Such reciprocal transplant experiments have been used to measure the extent of ecotypic differentiation and phenotypic plasticity (Nahum, Inbar, & Ne'eman, and Ben‐Shlomo., 2008), with the prediction that ecotypes adapted to one environment should change their phenotypes when placed in a novel environment, within their genetic constraints. Therefore, coupling reciprocal transplant with common garden experiments is critical because phenotypic plasticity of growth and defense traits in response to growing conditions can also generate clines, and such plasticity can obscure genetically based trait expression.

Here, we aim to measure the magnitude of ecotypic differentiation and plasticity in growth and defense traits for two unrelated plant species with similar geographical distribution along elevation gradients in the Alps (Supporting information Appendix S1: Figure S1). Specifically, we will address the following questions: (a) is there ecotypic differentiation in plant growth and defense‐related traits across an ecological gradient? (b) is there phenotypic plasticity in growth and defense‐related traits across different plant ecotypes, and (c) what is the magnitude of phenotypic plasticity for both growth and defense‐related traits along elevation gradients? To this end, we collected seeds of four populations of *Cardamine pratensis* (Brassicaceae) and six populations of *Plantago major *(Plantaginaceae); half of the populations originated from low elevation and the other half from high elevation (Supporting information Table S1). We reciprocally transplanted the high and low‐elevation ecotypes at both their elevation of origin or at the opposite elevation using two common gardens along a mountain transect and assessed variation in growth and defense (secondary metabolite) related traits.

**Figure 1:**
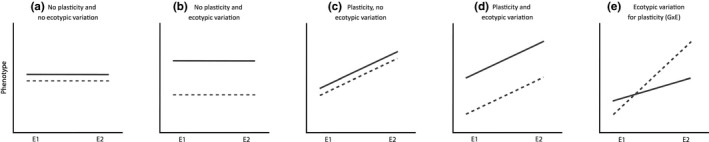
Theoretical framework for measuring ecotypic differentiation and phenotypic plasticity using reciprocal transplant experiments and reaction norms. The different panels represent all alternative scenarios. Line types represent different ecotypes, and E1 and E2 represent two different environments

Based on the theoretical framework shown in Figure 1 (Leggett, Brown, & Reece, 2014; Schlichting & Pigliucci, 1998), we expected five alternative scenarios: (a) no ecotypic variation or plasticity: traits remain constant across ecotypes and environments (Figure 1a). (b) ecotypic differentiation (ecotype effect only) with no plasticity: trait variation remains constant across elevations but different across ecotypes (Figure 1b). (c) plasticity without ecotypic differentiation (elevation effect only): both ecotypes show trait variations across different growing elevation, without significant difference between ecotypes (Figure 1c). (d) ecotypic effect accompanied by plasticity: different ecotypes exhibit differential values both from one another and at different growing elevation (elevation and ecotype effects) (Figure 1d), and finally (e) plasticity through ecotype by environment effect: the interaction of ecotype and elevation explains the traits value (elevation × ecotype effect) (Figure 1e). Overall, this study builds toward a better understanding of the ecological and evolutionary drivers of pathways mediating plant adaptation along ecological clines.

## 2 MATERIAL AND METHODS

### 2.1 Studied species

*Cardamine pratensis *is a rhizomatous perennial herb that grows in a variety of habitats including nutrient‐rich meadows, pastures, and forests and is common throughout Europe and in Central and Eastern Asia (Hultén & Fries, 1986). *C. pratensis *populations cover a wide elevation range, from sea level to about 1600 meters above sea level (Aeschimann, Lauber, Moser, & Theurillat, 2004), flowering from April to June. Flowers are self‐incompatible, and plants generally produce clonal offspring as new rosettes, especially under moist conditions (Lövkvist, 1956), and are considered hemicryptophyte (i.e. a long‐lived geophyte with overwintering green leaves). All *Cardamine pratensis *tissues, including leaves, contain glucosinolates (GLS), which, when in contact with myrosinases enzymes, are degraded into glucose and sulfate, along with various nitrile, isothiocyanate, and thiocyanate molecules that are toxic or deterrent to both herbivores and pathogens (Giamoustaris & Mithen, 1995; Hopkins, Ekbom, & Henkow, 1998; Kliebenstein, Pedersen, Barker, & Mitchell‐Olds, 2002; Lambrix, Reichelt, Mitchell‐Olds, Kliebenstein, & Gershenzon, 2001). Glucosinolates are often classified into three classes of compounds depending on their side‐chain: aliphatic, indole and aromatic, several of which have been shown to be effective against generalist and, to some extent, against specialist herbivores (Daxenbichler et al., 1991; Louda & Rodman, 1983; Montaut & Bleeker, 2011). Glucosinolates are known to vary quantitatively and qualitatively, across both individuals and populations of same species (Kliebenstein et al., 2001; Mauricio, 1998). In addition, phenotypic plasticity in GLS production has been previously observed in wild brassicaceous species (Agrawal, Conner, Johnson, & Wallsgrove, 2002). For instance, GLS profiles of *Boechera stricta* were strongly plastic, both among habitats and within habitats, and patterns of GLS plasticity varied greatly among genotypes (Wagner & Mitchell‐Olds, 2018).

*Plantago major *is a perennial (or facultatively perennial depending on environmental conditions) rosette‐forming herbaceous plant. As a poor competitor, *P. major* generally grows in ruderal areas, especially along paths or roadsides and near gateways where grass is short or absent (Warwick & Briggs, 1980). Native to Eurasia, *P. major* is a cosmopolitan species. It reproduces both sexually (self‐compatible wind pollinated) and asexually through rosette formation. Generally low genetic diversity among populations of *P. major *has been shown to favor ecotypic and phenotypic differentiation (Van Dijk, Wolff, & Vries, 1988; Halbritter, Billeter, Edwards, & Alexander, 2015; Warwick & Briggs, 1980). *P. major* can cover a very wide elevation range: from the sea level to alpine ecosystems up to 3,000 meters above sea level (Ren, Wang, Chen, & Zhu, 1999). *P. major *also produce notable amounts of secondary metabolites belonging to the class of cyclopentanoid monoterpenes, namely iridoid glycosides (IGs) and caffeoyl phenylethanoid glycosides (CPGs) (Pankoke, Buschmann, & Müller, 2013), which act as herbivore deterrents against generalist chewing insect (Fuchs & Bowers, 2004). IGs and CPGs display a relatively high degree of variation across plant tissues depending on plant population, plant phenology and environmental factors (Barton, 2008; Bowers & Stamp, 1993; Darrow & Bowers, 1999; Darrow & Deane Bowers, 1997; Miehe‐Steier, Roscher, Reichelt, Gershenzon, & Unsicker, 2015; Pellissier et al., 2014), and their production have been shown to display plasticity (Bowers & Stamp, 1992; Kuiper & Smid, 1985; Lotz & Blom, 1986).

### 2.2 Experimental design

*Cardamine pratensis* seeds were collected from two low‐elevation and two high‐elevation populations along two elevation gradients of the Jura Mountains in Switzerland in 2016. *Plantago major* seeds where collected from three low‐elevation and three high‐elevation population along three elevation gradients in the Swiss Alps during summer 2016 (Supporting information Table S1). Seeds were collected on randomly selected plants (*C. pratensis*, *n* = 6 plants/population; *P. major*, *n* = 10 plants/population) within a 100 m radius for each population.

While we acknowledge that we have not measured plasticity in the strict sense across genotypes, we here assumed that within a 100 m area, individuals are much more closely related than across populations. We, therefore, based all the analyses at the ecotypic level, assuming genetic clustering within populations. Seeds were thus pooled within populations. Harvested seeds were dried and kept at 4°C until the germination in Petri dishes lined with humid filter paper. One week after germination, 25 seedlings of *C. pratensis* per population (total of 100 plants) and 24 seedlings of *P. major* per population (total of 144 plants) were transplanted independently into plastic pots (13 cm width × 10 cm height) filled with mixture of 500 ml sieved soil compost (1 cm mesh size) (Ricoter, Aarberg, Switzerland) and sand (Neogard, Gontenschwil, Switzerland) in a 3:1 ratio. Plants were immediately transferred to a climate‐controlled chamber and kept on a 16 h/22°C ‐ 8 h/16°C day‐night cycle, and 50% relative humidity for 2 weeks, and received fertilizer twice a week until the beginning of the field experiment.

After two weeks of growth in the climate chamber, 25 *C. pratensis* plants per population and 24 *P. major *plants per population were equally distributed in two common gardens placed along the same mountain slope: La Neuveville (N: 47°06'84.28", E: 7°10'43.9", elevation: 450 m), and Chasseral (N: 47°07'03.36", E: 7°01'45", elevation: 1,600 m) at the beginning of July. The plants were left growing for a period of two months during summer 2017. The aim of a common garden is indeed to remove environmental variability for measuring genetic/ecotypic differentiation. By growing plants at two common garden elevations, we thus manipulated climatic conditions for measuring the extent of trait change (plasticity) due to changes in climatic regimes.

### 2.3 Plant growth‐related traits

After 8 weeks of growth in the field for both study species, aboveground biomass was separated from roots, oven‐dried at 40°C for 48 hr and weighed to determine their dry biomass. Furthermore, in *P. major *plants, two additional growth‐related traits were measured: (a) the chlorophyll content of the plant, which was measured as the average of three fully expanded leaves per plant using a SPAD‐502Plus chlorophyll meter (Konica Minolta (China) Investment Ltd), (b) the specific leaf area (SLA), which was measured as the one‐side area (calculated using ImageJ software) of the youngest fresh fully expanded leaf per plant divided by their oven‐dried (40°C for 48 hr) biomass (mm^2^ mg^−1^ DW) (Cornelissen et al., 2003). Higher SLA levels and chlorophyll content tend to positively correlate with potential relative growth rate, photosynthetic rate, or leaf nitrogen (N) across species (Garnier & Laurent, 1994; Poorter & Garnier, 2007). Generally, species in resource‐rich environments tend to have a higher SLA than those in resource‐poor environments (Garnier & Laurent, 1994; Poorter & Garnier, 2007).

### 2.4 Chemical analysis

For chemical analyses, sample preparation for each species followed different methods due to the different secondary metabolite extractions and analyses.

*Cardamine pratensis*: at the end of the experiment, one young fully expanded leaf was immediately frozen in liquid nitrogen and stored at −80°C; ground to powder using mortars and pestles in liquid nitrogen, and a 100 mg aliquot was weighed for GLS extraction. The extraction solvent (1.0 ml methanol: H_2_O: formic acid (70:29.5:0.5, v/v)) was added to the tubes along with 5 glass beads, shaken in a tissue lyser (Retsch GMBH, Haan, Germany) for 4 min at 30 Hz, and centrifuged at 26,560 *g* for 3 min. The supernatant was diluted 20 times with 70% methanol and transferred to an HPLC vial. Glucosinolate identification and quantification was performed using an Acquity ultra‐high pressure liquid chromatography (UHPLC) from Waters (Milford, MA) interfaced to a Synapt G2 quadrupole time‐of‐flight (QTOF) mass spectrometer from Waters with electrospray ionization, using the method as described in (Glauser, Schweizer, Turlings, & Reymond, 2012).

*Plantago major*: at the end of the experiment, one young fully expanded leaf was oven‐dried at 40°C for 48 hr prior being ground to powder using stainless steel beads in the tissue lyser. Then, 10 mg aliquots were weighed and 1.5 ml methanol was added to each tube along with 5 glass beads. The tubes were shaken 4 min at 30 Hz and centrifuged at 31,800 *g* for 3 min. The supernatant was diluted five times by adding 800 µl of MilliQ water to 200 µl of pure extract. Iridoid glycosides and CPGs were separated by UHPLC‐QTOF using an Acquity BEH C18 column from Waters (50 × 2.1 mm, 1.7 μm particle size) at a flow rate of 0.4 ml/min. The following gradient of water + formic acid 0.05% (phase A) and acetonitrile +formic acid 0.05% (phase B) was applied: 2%–9% B in 1.5 min, 9%–50% B in 3.5 min, 50%–100% B in 1.5 min, held at 100% B for 1.5 min, back to 2% B and held for 2.0 min. The column was maintained at 25°C. The injection volume was 1 μl. Detection was achieved in negative electrospray using deprotonated ions or formate adducts as quantification ions. Quantification ions and retention time of the two standards were: aucubin m/z 391.124 (formate adduct), retention time 1.17 min, and verbascoside m/z 623.198 (deprotonated ion), retention time 3.16 min. Absolute amounts of IGs and CPG were determined by external calibration using five standard solutions of aucubin at 0.2, 0.5, 2, 5 and 10 μg/land verbascoside at 0.2, 0.5, 2, 5 and 20 μg/ml. Concentrations were normalized to plant weight and expressed as μg/mg. Other Iridoid glycosides and caffeoyl phenylethanoid glycosides were putatively identified based on their retention time and chemical formula by comparing them to previous detection in *P. major *or in species of *Plantago *genus (Rønsted, Göbel, Franzyk, Jensen, & Olsen, 2000) and database (Dictionary of Natural Products, CRC Press, USA, version 6.1. on DVD) containing information on known IGs and CPGs and quantified as aucubin or verbascoside equivalents. Iridoid glycosides named with the code IG followed by numbers (Supporting information Figure S2) represent molecular formula corresponding to potential IG for which several isomers exist in the literature and thus cannot be unequivocally annotated.

### 2.5 Herbivore bioassay

To measure plant resistance against insect herbivores (defined as the effect of plant defense traits on herbivore performance (Karban & Baldwin, 1997)), we used the generalist herbivore, *Spodoptera littoralis* (Lepidoptera: Noctuidae; obtained from Syngenta, Stein AG, Switzerland). *S. littoralis* is known to feed on species belonging to more than 80 families of plants (Brown & Dewhurst, 1975), and is widely used for performing plant resistance bioassays. Here, we consider caterpillar weight gain during a fixed time period as an integrative measure of plant resistance, reflecting the global defensive state of the plant (i.e. both physical and chemical traits).

Newly hatched larvae were reared on a corn‐based artificial diet for 7 days before the beginning of the bioassay. Immediately after removal of plants from the field, both plant species were placed in a climate‐controlled chamber (24/18°C, 16/8 hr, day/night regime, and 55% R.h.) to homogenize the condition for herbivores feeding on both species during the bioassay. For *C. pratensis, *one fully expanded new leaf from 12 plants per population that grew at the two elevation common gardens (*n* = 48) was cut and placed in a Petri dish lined with a moist filter paper. One 7‐day old *S. littoralis* larva was added to each petri dish. For *P. major*, we instead performed a whole plant bioassay. We placed two 7‐day old *S. littoralis* larvae on 24 plants per ecotype/population that were growing at the two elevation common gardens (*n* = 96). Plants were covered with nylon nets to avoid escaping of caterpillars. After five days of herbivory for *C. pratensis* and three days for *P. major*, the insects were retrieved from individual Petri dishes and plants, respectively and their weights were measured and recorded. We calculated larval weight gain using the formula ln (final weight − initial weight) For *P. major*, larval weight gain was averaged across the two caterpillars on each plant. Lower weight gains indicate that plants are more resistant (Humphrey et al., 2018).

### 2.6 Statistical analyses

All statistical analyses were performed within the R environment (R Development Core Team, 2017). For chemical data, we calculated the sum of glucosinolate compounds (GLS total) for *C. pratensis* and the sum of iridoid glycosides (IGs total) and caffeoyl phenylethanoid glycosides (CPGs total) for *P. major*, as well as a measure of chemical diversity for both plant species using the Shannon‐Weaver diversity indices (Hill, 1973) with the *diversity* function in the *vegan *package in R (Oksanen et al., 2017).

To measure the interactive effects of elevation of origin and elevation of growth on plant growth and defense traits, we used two‐way ANOVAs, including transplant sites (high and low), elevation ecotypes (high and low), and their interaction as fixed factors. We also included the term population nested within elevation ecotypes in the model to assess variability across populations within a given elevation of origin. The response variables were aboveground biomass (AG biomass), larval weight gain, total GLS, total indole, total aliphatic, and chemical diversity for *C. pratensis*, and AG biomass, chlorophyll content, SLA, larval weight gain, total chemistry, total IGs, total CPGs and chemical diversity for *P. major*. All chemical traits were log‐transformed prior analyses to meet normality and homoscedasticity assumptions. A significant effect of site of growth (i.e. elevation) would indicate a plastic response to different environmental conditions. A significant effect of ecotype would indicate differentiation in traits among populations belonging to different ecotypes. A significant effect of population would indicate differentiation in traits among populations. A significant elevation × ecotype term would indicate ecotype‐specific plastic response for a given trait depending on the growing elevation (Figure 1).

To address the multivariate nature of plant secondary compound blends, we also ran a full‐factorial model including the individual secondary metabolites abundance matrix as response variable and plant ecotype and elevation as factors using permutational analysis of variance (PERMANOVA) with the *adonis* function in the *vegan* package in R (Oksanen et al., 2017). We also included plant biomass as covariate to control for potential direct effect of plant size (i.e. total aboveground biomass) on plant chemistry (Züst, Rasmann, & Agrawal, 2015). The Bray–Curtis metric was used to calculate a dissimilarity matrix of all compounds among samples for the PERMANOVA. We visualized ecotypic differentiation of the secondary metabolites using an NMDS ordination analysis of the chemical compounds based on Bray Curtis distance using the *vegan* package in R (Oksanen et al., 2017).

Finally, we calculated and visualized the magnitude of plasticity of plant growth and defense related traits when plants were placed in the elevation opposite to their elevation of origin. We calculated the standardized effect sizes (SES) for all traits as standardized mean difference (SMD) = ((*µ*1 – *µ*2)/*s*) (*µ*1 = mean trait value at opposite elevation growing site, *µ*2 = mean trait value at elevation of origin, *s* = standard deviation) using the *effsize* function (implemented with the *cohen.d* metrics) in the *effsize* package in R (Torchiano, 2017). Using effect sizes allows us allows us to compare different traits within the same analysis. The resulting figure constructed based on effect size represents the plastic response of traits, ecotype × environment effects, as well as the magnitude of responses. A 95% of confidence interval bar that deviates from zero shows a significant trait change when growing at the opposite elevation (Nakagawa & Cuthill, 2007). On the other hand, while comparing two ecotypes (high and low), if one deviates from zero but not the other one, it would indicate ecotype × elevation of growth effects.

## 3 RESULTS

### 3.1 Plant growth‐related traits

**Figure 2:**
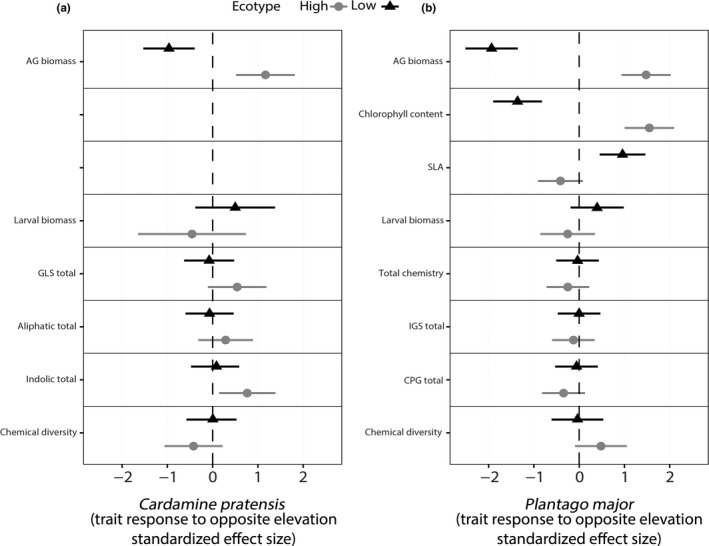
Cohen's d standardized effect sizes (±95% CI) for the influence of growing at opposite elevations of origin on plant growth and defense related traits, for high and low‐elevation ecotypes of *C. pratensis* (a) and *P. major *(b)

**Figure 3:**
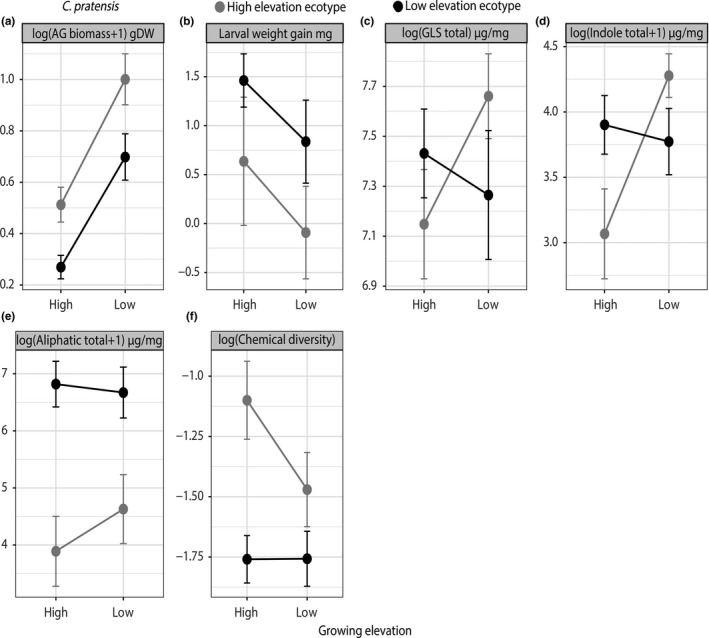
Reaction norms of *C. pratensis* ecotypes for growth (a), larval weight gain (b) and defense (c, d, e, f) traits. Mean phenotypic values (mean ±1 *SE* for each elevation ecotype) are represented in black (low‐elevation ecotypes) or gray (high‐elevation ecotypes) across two contrasting growing elevations (high or low)

**Figure 4:**
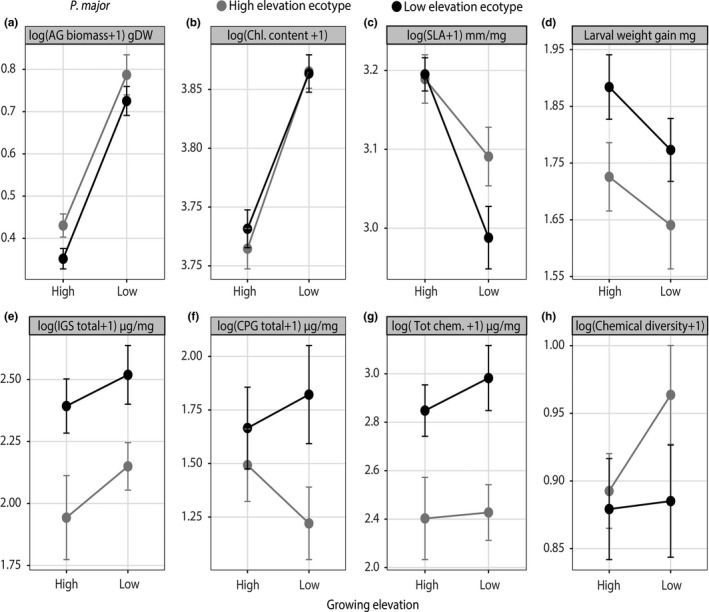
Reaction norms of *P. major* ecotypes of growth traits (a, b, c), larval weight gain (d) and defense traits (e, f, g (total chemistry), h). Mean phenotypic values (mean ±1 *SE* for each elevation ecotype) are represented in black (low‐elevation ecotypes) or greay (high‐elevation ecotypes) across two contrasting growing elevations (high or low)

For both species, we observed phenotypic plasticity and ecotypic differentiation in aboveground (AG) biomass, through significant effects of both ecotype (*p* < 0.001; *C. pratensis, p* = 0.03; *P. major*) and elevation (high or low‐elevation growing sites) (*p* < 0.001; *C. pratensis, p* < 0.001; *P. major*) (Figures 2, 3, 4; Table 1). We observed that AG biomass of high‐elevation ecotypes increased by 49% (SMD = 1.17) for *C. pratensis* and by 45% (SMD = 1.48) for *P. major* when growing at low elevation, while AG biomass of low‐elevation ecotypes’ decreased by 61% (SMD = −0.96) for *C. pratensis* and by 51% (SMD = −1.93) for *P. major *when growing at high elevation (Figures 2, 3, 4; Table 1). Furthermore, our results indicated that high‐elevation ecotypes produced 38.5% and 12% more AG biomass than low‐elevation ecotypes in *C. pratensis *and *P. major*, respectively. In addition, in *P. major* leaf chlorophyll content and SLA showed plasticity through growing elevation effect (*p* < 0.001), with the latter also showing marginal ecotype × environment effect (*p* = 0.09). Specifically, we observed that chlorophyll content of high‐elevation ecotypes increased by 4.1% (SMD = 1.55) when placed at low elevation, and low‐elevation ecotypes had 3.4% (SMD = −1.36) less chlorophyll content when growing at high elevation (Figures 2b, 4; Table 1). Moreover, SLA of low‐elevation ecotypes significantly increased by 6.6% (SMD = 0.96) when growing at high elevation (Figures 2b, 4; Table 1).

**Table 1:** Two‐way ANOVA results, indicating interactions between the effects of high and low‐elevation ecotypes and elevation of growth (in two common garden sites) on growth and defense traits

Plant species	Response variable	Factor	*df*	Mean SQ	*F* value	*p* value
*C. pratensis*	AG biomass	Ecotypes	1	2.15	14.59	<0.001***
		Population	2	0.09	0.64	0.53
		Elevation	1	5.22	35.41	<0.001***
		Ecot *	1	0.02	0.14	0.7
	Total GLS	Ecotypes	1	0.16	0.17	0.7
		Population	2	4.71	5	0.009**
		Elevation	1	0.38	0.40	0.5
		Ecot *	1	3.21	4	0.07†
	Total indole	Ecotypes	1	0.6	0.38	0.5
		Population	2	2.59	1.63	0.2
		Elevation	1	5.46	3.44	0.07†
		Ecot *	1	11.45	7.22	0.009**
	Total aliphatic	Ecotypes	1	154.86	23.40	<0.001***
		Population	2	56.78	10.41	<0.001***
		Elevation	1	1.52	0.28	0.6
		Ecot *	1	4.72	0.87	0.4
	Chemical diversity	Ecotypes	1	4.69	12.33	<0.001***
		Population	2	0.72	1.89	0.2
		Elevation	1	0.59	1.55	0.22
		Ecot *	1	0.91	2.4	0.12
	Larval weight gain	Ecotypes	1	7.73	4.38	0.04*
		Population	2	0.06	0.04	1
		Elevation	1	4.03	2.28	0.1
		Ecot *	1	0.02	0.01	0.9
*P. major*	AG biomass	Ecotypes	1	0.18	4.75	0.03*
		Population	4	0.1	2.47	0.047*
		Elevation	1	4.63	118.88	<0.001***
		Ecot *	1	0.004	0.09	0.8
	Chlorophyll content	Ecotypes	1	0.0008	0.1	0.8
		Population	4	0.02	2.28	0.06†
		Elevation	1	0.68	81.79	<0.001***
		Ecot *	1	0.003	0.32	0.6
	SLA	Ecotypes	1	0.07	1.89	0.2
		Population	4	0.08	2.38	0.05†
		Elevation	1	0.81	23.14	<0.001***
		Ecot *	1	0.1	2.78	0.09†
	Total IG	Ecotypes	1	4.26	12.65	<0.001***
		Population	4	2.34	6.97	<0.001***
		Elevation	1	0.7	2.07	0.2
		Ecot *	1	0.04	0.1	0.7
	Total CPGs	Ecotypes	1	3.51	4.1	0.04*
		Population	4	2.14	2.49	0.04*
		Elevation	1	0.09	0.11	0.7
		Ecot *	1	1.1	1.28	0.3
	Total chemistry	Ecotypes	1	6.2	14.78	<0.001***
		Population	4	1.4	3.33	0.01*
		Elevation	1	0.0.16	0.37	0.5
		Ecot *	1	0.08	0.18	0.7
	Chemical diversity	Ecotypes	1	0.05	1.66	0.2
		Population	4	0.09	3.11	0.02*
		Elevation	1	0.04	1.28	0.3
		Ecot *	1	0.02	0.76	0.4
	Larval weight gain	Ecotypes	1	0.2	8,66	0.004**
		Population	4	0.36	14.78	<0.001***
		Elevation	1	0.1	4.07	0.047*
		Ecot *	1	0.0003	0.01	0.9

Note

Signif. Codes for *p‐value*: 0 “^***^” 0.001 “^**^” 0.01 “^*^” 0.05 “†” 0.1.

### 3.2 Plant chemical defenses and resistance

The glucosinolate profiles of *C. pratensis* leaves consisted of six GLS compounds (two aliphatic, three indoles and one aromatic), and the secondary metabolites profile of the *P. major* leaves consisted of 13 IGs and 3 CPGs compounds (Supporting information Figure S2).

**Figure 5:**
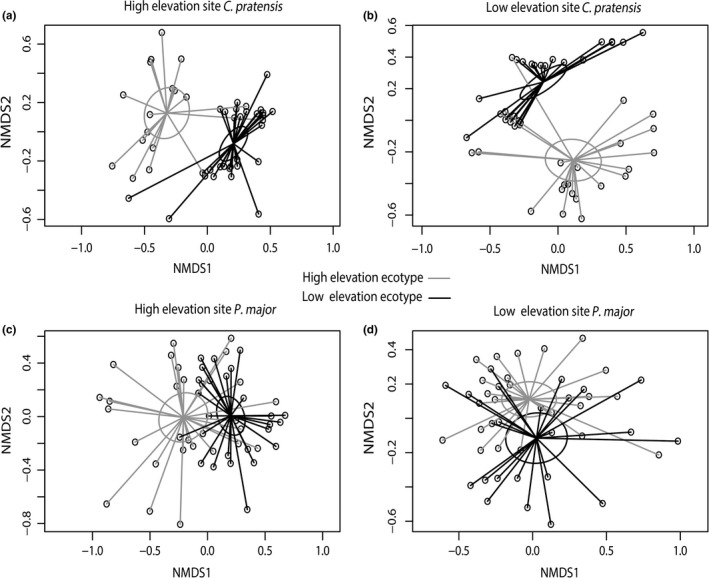
Non‐metric multidimensional scaling (NMDS) plot of *Cardamine pratensis* plant ecotype of (a: high elevation and b: low‐elevation common gardens) and *Plantago major *(c: high elevation and d: low‐elevation common gardens). Distance matrices were generated using secondary metabolite (glucosinolates in *C. pratensis* and *iridoid glycosides and *caffeoyl phenylethanoid glycosides for *P. major*) concentrations and diversity. The 95% confidence interval ellipses are represented based on the two elevation ecotypes (high‐elevation ecotype in gray and low‐elevation ecotype in black). Stress values:  (a) and (b) = 0.12, (c) and (d) = 0.2, K = 2

In *C. pratensis*, we observed phenotypic plasticity in total indole GLS (ecotype × environment effect, *p* = 0.009), where the total indole GLS concentration of high‐elevation ecotypes significantly increased at the low elevation by 28% (SMD = 0.77), while indole GLS of low‐elevation ecotypes does not vary (Figures 2a, 3; Table 1). Low‐elevation ecotypes produced 37% more aliphatic GLS than high‐elevation ecotypes, and high‐elevation ecotypes showed 25% more chemical diversity than low‐elevation ecotypes (Figure 3, Table 1). Furthermore, the PERMANOVA (Supporting information Table S2) showed that the abundance and chemical diversity of GLS were globally different across elevation ecotypes (elevation ecotype effect, *F = *41.85; *p* = 0.001) but there was no elevation ecotype × elevation of growth effect (Figure 5a,b). We found ecotypic effect in insect weight gain; larvae on low‐elevation ecotypes grew 81% more compared to high‐elevation ecotypes (Table 1, Figure 3b). Finally, we also found significant population‐level effects for several traits (See Supporting information Figure S3 and Table 1), indicating that local differentiation in trait expression is also influenced by adaptation to different mountain transects.

In *P. major,* in terms of absolute compound quantities, low‐elevation plants produced 17% more compounds in total, 17% more IGs, and 22% more CPGs (Figure 4, Table 1). The PERMANOVA (Table S2) revealed a plant ecotypic effect (elevation ecotype effect, *F* = 4.5; *p* = 0.001) and a growing elevation effect (*F* = 3.55; *p* = 0.006) (Figure 5c,d) in the abundance and diversity of secondary metabolites in *P. major*. Additionally, we found that abundance of the total chemistry and diversity of the compounds were significantly affected by the AG biomass of *P. major *(*F* = 8.6; *p* = 0.001). For *P. major, *we also observed signify cant effects of population‐level effect on all the measured traits (marginal for SLA and chlorophyll content) (Supporting information Figure S4 and Table 1). Finally, we also found ecotypic differentiation for *S. littoralis* larval weight gain (Figure 4d*, *Table 1): larvae on low‐elevation ecotypes grew 8% more than on high‐elevation ecotypes.

## 4 DISCUSSION

The major aim of this study was to elucidate on the variable responses of growth versus defense related traits using common gardens of plant ecotypes growing at different elevations. We observed ecotypic differentiation accompanied by plasticity in growth‐related traits, while we mainly observed ecotypic differentiation for defense traits for both *P. major* and *C. pratensis*. Below, we outline the potential causes for such divergence along elevation gradients.

### 4.1 Plant biomass accumulation

Plasticity can be visualized as a change in the slope of the reaction norm between the ecotype at the elevation of origin and the same ecotype growing at opposite elevation (Doughty, 1995; Gotthard et al., 1995). In this regard, for both species, plant growth‐related traits (AG biomass, leaf chlorophyll content and SLA) showed plasticity (Figures 2, 3a, 4a,b, c). Our results compliment other findings where the combination of ecotypic differentiation and phenotypic plasticity in growth‐related traits such as biomass and flower size was shown for invasive species at their invasive range (Martín‐Forés et al., 2017). More specifically, we observed that in both species, the AG biomass across both ecotypes was higher at low‐elevation growing sites and lower at high‐elevation growing sites (Figures 3a, 4a). Higher AG biomass production of both ecotypes at low‐elevation growing site comes as no surprise, given the growing conditions at low‐elevation are warmer and more favorable than at high elevation. Two reasons have been put forward for plants to reduce growth at high elevation. First, a decrease in the general metabolic activity as a function of colder temperature inhibits photosynthetic rate and biomass production (Boyer, 1982). Second, it has been proposed that because plants growing at higher elevations typically receive direct sunlight and higher ultraviolet radiation, and ultraviolet radiation destroys the auxins content at the apical shoots, they tend to grow much slower than lowland plants (Keller, Stahlberg, Barkawi, & Cohen, 2004). Furthermore, as both *C. pratensis* and *P. major* are perennial species, it could be argued that high‐elevation ecotypes accumulated higher AG biomass than low‐elevation ecotypes once placed in more favorable low‐elevation conditions to compensate for the next year's growing season, when they would have to allocate more resource to flower and seed production. Such a scenario should be less likely for low‐elevation plants growing at their elevation of origin. However, we make this argument with caution for *P. major*, since it is a facultative perennial plant.

Interestingly, we also observed that high‐elevation ecotypes of both species always produced more biomass than low‐elevation ecotypes (Figures 3a, 4a). This is somewhat surprising, since we expected alpine plants to grow smaller in harsher and colder environments (Atkin & Day, 1990; Körner, 2003). Plant size is negatively correlated with extremely cold temperatures (Squeo, Rada, Azocar, & Goldstein, 1991) and as a consequence, generally decreases with elevation (Körner, 2003). Plants adapted to high elevation, where growing season is short, should favor fast biomass accumulation (Körner, 2016). For instance, plants growing in colder conditions typically exhibit greater photosynthetic and respiratory capacities than their warmer‐grown counterparts (Atkin, Loveys, Atkinson, & Pons, 2006). Therefore, high‐elevation ecotypes could benefit from faster development and high rates of metabolism (Körner, 2016), and, at equal growing conditions (same soil) and during the same growing timeframe, have actually accumulated more biomass than their low‐elevation counterparts.

Finally, we also want to note that because we worked at the ecotypic level, one might argue that the plastic response we observed in growth‐related traits might be driven by genotypic differences within each population. In other words, if a population is highly genetically differentiated, a random sampling would result in more likely piking highly plastic genotypes, which would drive the overall population mean change. If this were the case, larger (in our case lowland) populations should have shown higher levels of plasticity overall, but this was not the case (see Supporting information Figures S3 and S4).

### 4.2 Plant chemical defenses and resistance

We observed ecotypic differentiation across most plant defense and resistance measures in both species. First, the ordination showed ecotypic differentiation for the overall secondary metabolite blend for both species (see Supporting information Table S2 and ecotypic segregation in the NMDS plot in Figure 5) despite the pattern of production (increase or decrease in concentration). Similarly, aliphatic GLS, chemical diversity, total IGs, total CPGs, and larval weight also clearly showed ecotypic differentiation for both species. (Figures 3e,f, 4d,e,f). Generally, regardless of the growing elevation, low‐elevation ecotypes produced more chemical defenses (Figures 3c, 4g). These results are in line with other findings showing cold temperature‐driven suppression of plant secondary metabolites (Pellissier et al., 2014), and a general decrease in secondary metabolite production at high elevation (Kergunteuil et al., 2018). However, a decrease in secondary metabolite production in high‐elevation ecotypes could also be attributed to a decrease in herbivory pressure at high elevation. To date, we have no data that allows disentangling biotic and abiotic effects of defense decline at high elevation, but likely both synergistically interact for selecting such a chemical phenotype (Pellissier et al., 2014).

Interestingly, however, indole GLS showed no ecotypic differentiation: high‐elevation ecotypes produced more of these compounds when placed at low‐elevation (see ecotype × environment effect in Table 1). Unlike aliphatic GLS, for which induction has been rarely observed (Koritsas, Lewis, & Fenwick, 1991; Li, Kiddle, Bennett, & Wallsgrove, 1999), induction of indolic GLS has been widely documented in several systems (Agrawal, Strauss, & Stout, 1999; Doughty, Kiddle, Pye, Wallsgrove, & Pickett, 1995; Griffiths, Birch, & Macfarlane‐Smith, 1994; Moyes, Collin, Britton, & Raybould, 2000; Raybould & Moyes, 2001; Siemens & Mitchell‐Olds, 1998), including in the closely related *Cardamine hirsuta* (Bakhtiari, Glauser, & Rasmann, 2018). In addition, indole GLS have been previously shown to be strongly influenced by environmental factors, suggesting favorable selection pressures for plasticity in this class of secondary metabolites. If plasticity is a means of saving energy (Bidart‐Bouzat, Mithen, & Berenbaum, 2005; Traw, 2002), this could indicate that the production of indole GLS might be more costly than the production of other GLS in *C. pratensis *at high elevation. On the other hand, it might also indicate that temperature dictates indole GSL production more than other classes of GSLs, because indole GSL compounds are intrinsically more inducible. In other words, we could imagine a scenario in which energy‐saving plasticity of induction has evolved in response to variable herbivory pressure (i.e. optimal defense hypothesis Zangerl and Rutledge (1996)) (Agrawal et al., 2002; Humphrey et al., 2018; Wagner & Mitchell‐Olds, 2018), and it has been retained during range expansion toward higher elevations. Therefore, plasticity in defense‐related traits is a reflection of both biotic and abiotic environmental conditions that affect the expression of defenses. Conversely, the lack of plasticity in the majority of defense related traits in our study could be because the benefits of plasticity could not outweigh the costs affiliated with high herbivore pressure earlier in the season, or other potential costs of defense plasticity. For example, indolic GLS did not show plasticity, in contrast to non‐indolic GLS, in *Cardamine cordifolia *plants growing in shaded‐common gardens, that are characterized by low herbivory (Humphrey et al., 2018). In contrast to our results, Humphrey et al. (2018) also found plasticity in larval weight gain of a specialist herbivore (*Scaptomyza nigrita*).

Detailed analysis of the effect sizes (SESs) between growth and defense related traits in *C. pratensis* (Figure 2a) indicates that the plasticity displayed by high‐elevation ecotypes is higher for AG biomass (very large SES) (Cohen, 1988) compared to indolic GLS production (large SES). In *P. major* (Figure 2b) the magnitude of plastic responses in all growth‐related traits were also very large, compared to the non‐significant plastic responses for all defense‐related traits (except for some the individual compounds, Supporting information Figure S2). Nevertheless, the lack of plastic response to elevation in defense‐related traits does not completely discard the potential for plastic responses in chemical defenses. The environmental effects of growing elevation could influence plant chemistry at any time throughout the growing season; since chemistry was measured only at the end of the field season, plasticity in expression of such traits could have disappeared by the end of the season. Moreover, the phytohormone activation machinery underlying expression of chemical defenses in response to herbivory is a very fast process (Mousavi, Chauvin, Pascaud, Kellenberger, & Farmer, 2013). In contrast, the detection of the potential plastic responses in plant defense to abiotic stimuli might be masked by the time‐dependency of the growing season (Anderson, Lee, & Mitchell‐Olds, 2011). Additionally, two studies on *C. cordifolia* and *P. lanceolata* showed phenological variation in plant tissue GLS and IGs content, respectively (Darrow & Deane Bowers, 1997; Rodman & Louda, 1984). Therefore, ontogeny should also be addressed when measuring plasticity, since plants have been shown to express different levels of plasticity in defense traits as they grow.

## 5 CONCLUSIONS

Few studies have assessed phenotypic variation of plant growth versus defense traits in response to contrasting environments. Here, we documented that plant growth traits displayed strong ecotypic differentiation accompanied by plasticity, but, in contrast, we found little support of phenotypically plastic defense and resistance traits in response to different growing habitat across steep elevation gradients. Future research on similar systems would require coupling the observed effects on plant phenotypes with genetically‐explicit fitness measurements and selection gradient analyses in order to disentangle the fitness benefits of phenotypic plasticity versus fixed ecotypic differentiation at the population level.

## CONFLICT OF INTEREST

The authors declare no conflict of interest.

## AUTHOR CONTRIBUTIONS

MB, LF and VC performed the experiment, collected and analyzed the data. SR conceived the study, analyzed the data. GG assisted with chemical analysis. MB, LF and SR wrote the manuscript.

## Data Availability

The data associated with this publication are deposited at Dryad data repository. Provisional DOI: https://doi.org/10.5061/dryad.4b14m4r. Data files title: Growth‐, resistance‐, and chemical‐related trait measurement of *C. pratensis *and *P. major plant*.
