# Supplementary material for: Variable effects on growth and defense traits for plant ecotypic differentiation and phenotypic plasticity along elevation gradients
Source: Ecol Evol. 2019 Feb 27;9(7):3740–55. doi: 10.1002/ece3.4999 (PMC6468067; doi:10.1002/ece3.4999)
Supplement: Supplementary file 1 [file ECE3-9-3740-s001.docx]

**Appendix**

**Table A1.** Coordinates of the plant populations.

| **Plant specie** | **Site** | **Altitude (m.a.s.l.)** | **N** | **E** |
| --- | --- | --- | --- | --- |
| *C. pratensis* | A. Chasseral (BE, Jura mountain ) | 1607 | 47°07'38.0" | 7°02'40.1" |
|  | B. Chasseron (VD, Jura mountain ) | 1607 | 46°51'21.6" | 6°32'34.8" |
|  | C. Cortaillod (NE, Jura) | 484 | 46°55'50.5" | 6°49'56.6" |
|  | D. Cheseaux-Noréaz (VD, Jura) | 476 | 46°46'55.2" | 6°40'19.0" |
| *P. major* | A. Somprei (TI, south Alps) | 1880 | 46°30′29.273″ | 8°46′33.515″ |
|  | B. Aminona (VS, Alpes) | 1778 | 46°20′15.029′′ | 7°32′6.388′′ |
|  | C. Morcles (VD, Prealps) | 1794 | 46°12′53.623′′ | 7°3′3.421′′ |
|  | D. Mairengo (TI, south Alps) | 880 | 46°29′13.329″ | 8°47′33.239″ |
|  | E. Leuk (VS, Alpes) | 657 | 46°18′24.429″ | 7°40′18.350′′ |
|  | F. Lavey (VD, Prealps) | 521 | 46°11′53.302′′ | 7°1′34.758′′ |

# Table A2. Two-way PERMANOVA results, based on Bray-Curtis dissimilarities (999 permutations) of *Cardamine pratensis* and *Plantago major* secondary metabolite abundance and diversity, sampled across elevation-ecotype (Ecotype) and growing elevation (Elevation). Plant aboveground biomass (AG biomass) is included as covariate in the analysis.

| **Plant species** | **Factor** | **Df** | **Mean SQ** | **pseudoF-value** | **R^2^** | **P value** |
| --- | --- | --- | --- | --- | --- | --- |
| *C. pratensis* | Ecotypes | 1 | 7.61 | 41.85 | 0.31 | **0.001***** |
|  | Elevation | 1 | 0.23 | 1.24 | 0 | 0.28 |
|  | Ecot *Elev | 1 | 0.26 | 1.48 | 0.01 | 0.18 |
|  | AG biomass (covariate) | 1 | 0.40 | 2.24 | 0.01 | 0.07 |
|  | Residuals | 88 | 0.18 |  | 0.65 |  |
| *P. major* | Ecotypes | 1 | 0.84 | 4.5 | 0.04 | **0.001***** |
|  | Elevation | 1 | 0.66 | 3.55 | 0.03 | **0.006**** |
|  | Ecot *Elev | 1 | 0.28 | 1.50 | 0.01 | 0.173 |
|  | AG biomass (covariate) | 1 | 1.60 | 8.60 | 0.08 | **0.001***** |
|  | Residuals | 95 | 0.19 |  | 0.84 |  |

Signif. Codes for *p-value*: 0 ‘***’ 0.001 ‘**’ 0.01 ‘*’ 0.05 ‘.’ 0.1 ‘ ’ 1


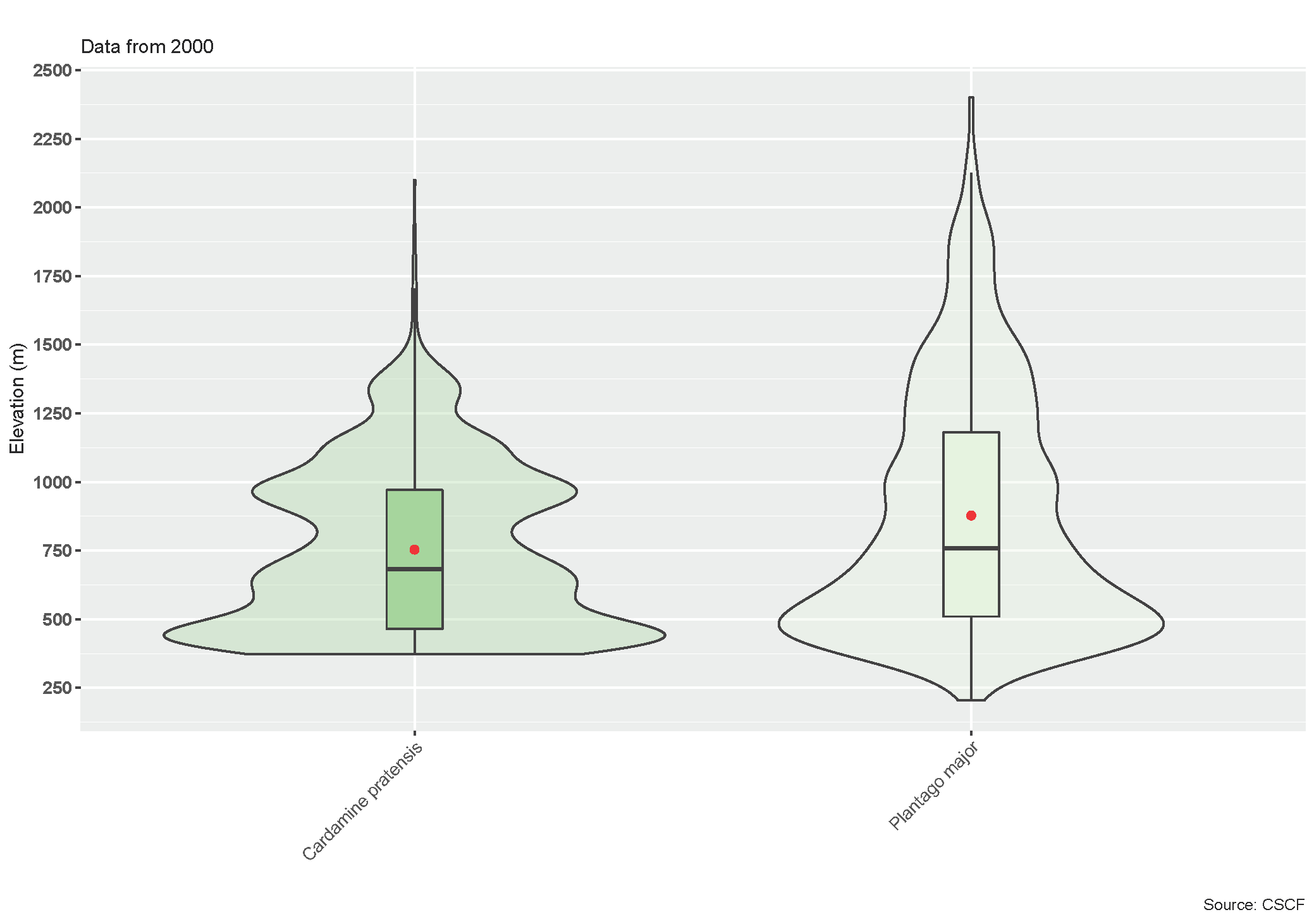


**Fig. A1.** Distribution of *Cardamine pratensis* and *Plantago major* along elevation gradients in Switzerland. Data obtained from www.infoflora.ch. Violin plots represent the scaled abundance of observations at different elevations, and indirectly represent species abundances at different elevations.


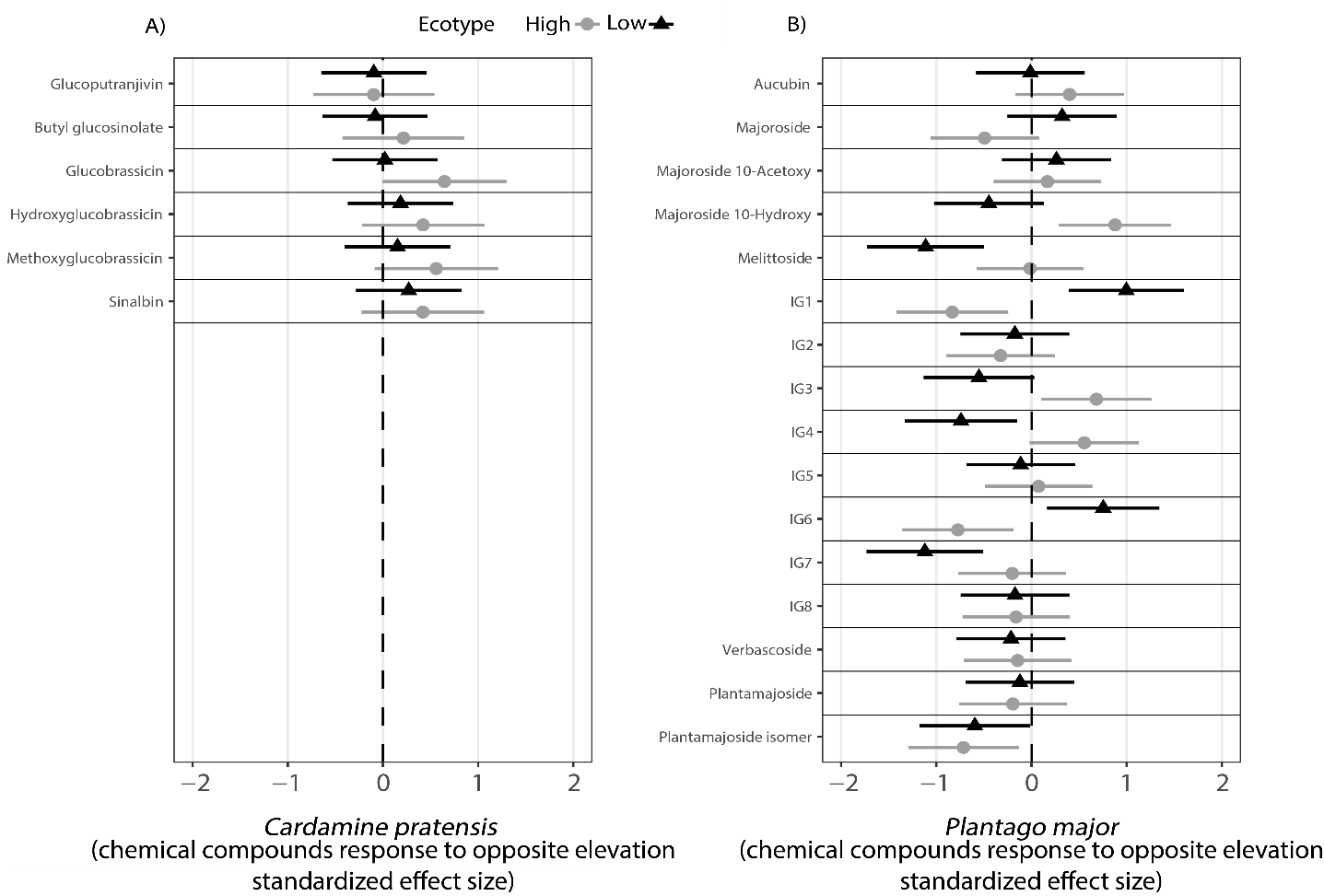


**Fig. A2.** Effect sizes for the influence of non-native growing elevation on plant secondary metabolite compounds for high and low elevation populations of *C pratensis* (A) and *P. major* (B). Effects are standardized effect size (SES) with 95% confidence limits.


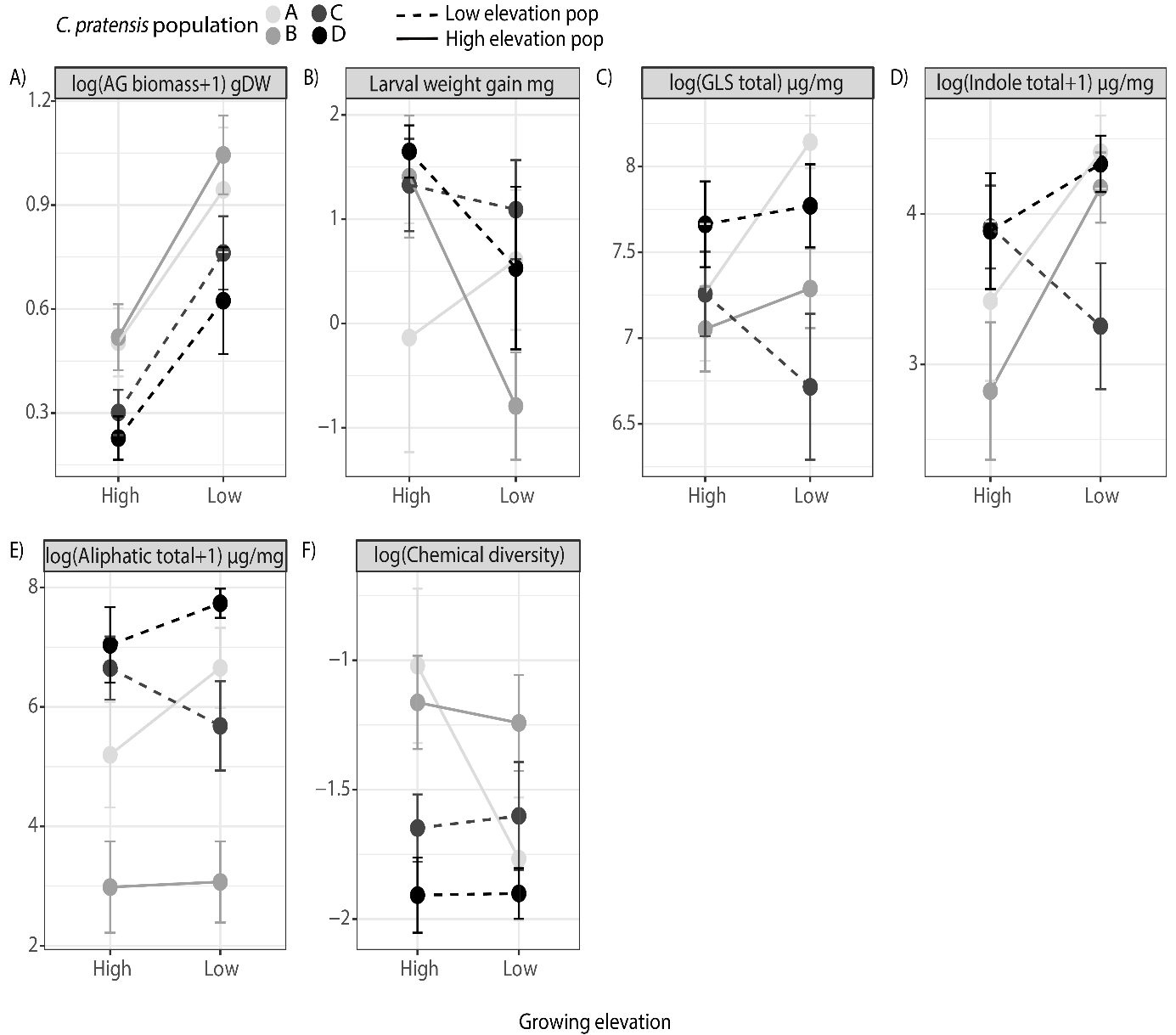


**Fig. A3.** Reaction norms of *Cardamine pratensis* populations of growth (A), resistance (B) and defence (C, D, E, F) traits. Mean phenotypic values (mean ± 1 s.e. for each elevation population) are represented in black (low elevation populations) or grey (high elevation populations) across two contrasted growing elevations (high or low).


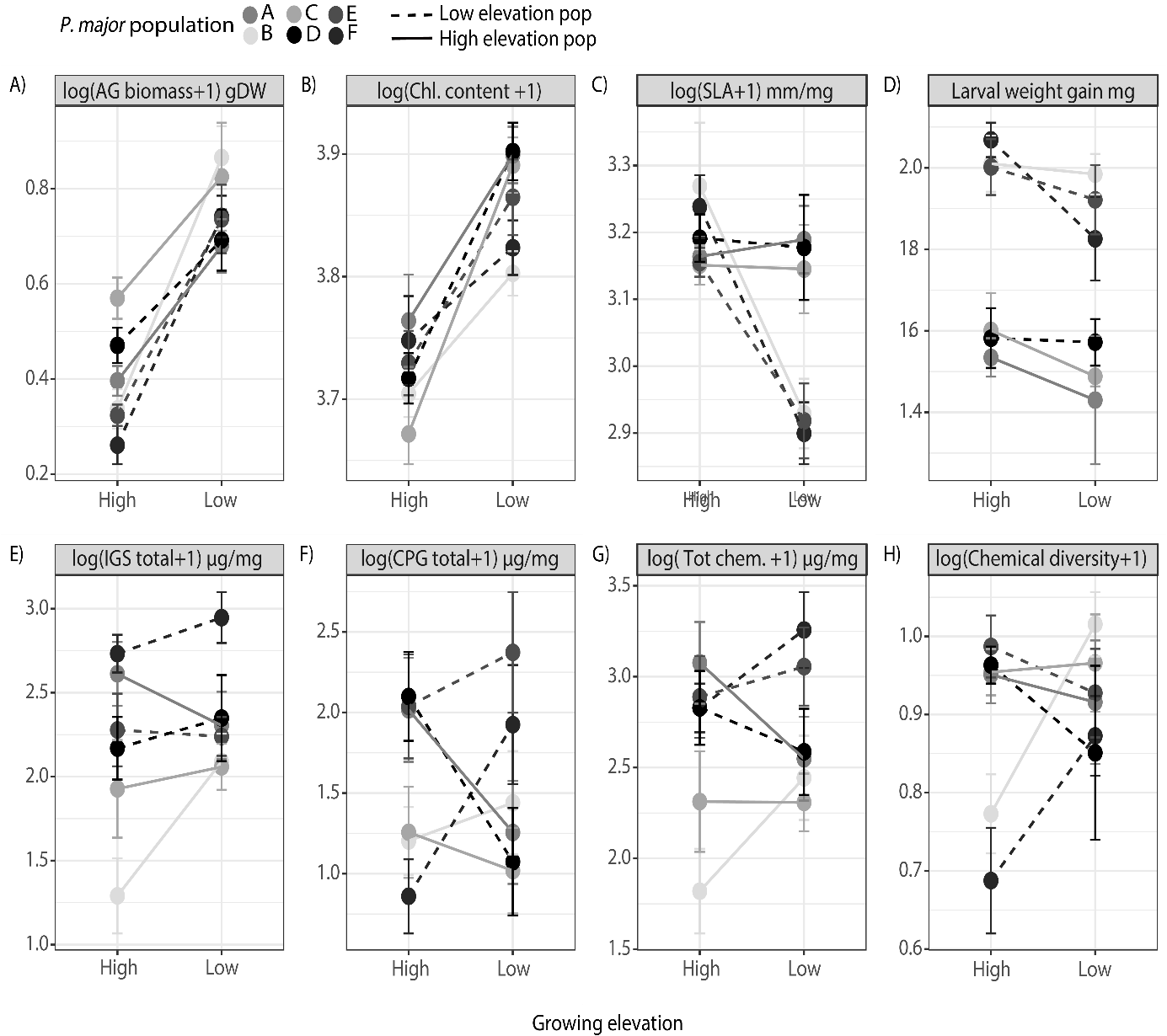


**Fig. A4.** Reaction norms of *P. major* populations of growth (A, B, D), resistance (D) and defence (E, F, G (total chemistry), H) traits. Mean phenotypic values (mean ± 1 s.e. for each elevation population) are represented in black (low elevation populations) or grey (high elevation populations) across two contrasted growing elevations (high or low).
